# ^18^F-FDG PET/CT imaging for aggressive melanotic schwannoma of the L3 spinal root: A case report

**DOI:** 10.1097/MD.0000000000024803

**Published:** 2021-02-26

**Authors:** Xun-Ze Shen, Wei Wang, Zhou-Ye Luo

**Affiliations:** a PET/CT Center, Shaoxing People's Hospital, the First Affiliated Hospital of Shaoxing University, 568 Zhongxing North Rd, Shaoxing City; b Wei Wang, Department of Pathology, Zhejiang Provincial People's hospital, Hangzhou City, Zhejiang Province, China.

**Keywords:** aggressive, fluorodeoxyglucose positron emission tomography/computed tomography, liver, melanotic schwannoma, spinal

## Abstract

**Rationale:**

Melanotic schwannoma (MS) is an unusual variant of a nerve sheath neoplasm that accounts for less than 1% of all primary peripheral nerve sheath tumors. Fluorine-18 fluorodeoxyglucose positron emission tomography/computed tomography (^18^F-FDG PET/CT) has unique value in detecting malignant MS lesions. To date, only 4 cases of MS with hepatic metastasis have been reported. Herein, we report the fifth case, which is the first reported patient with MS of Asian ethnicity with hepatic metastasis.

**Patient concerns:**

A 29-year-old woman with a 1-day history of backache was admitted to our hospital. PET/CT showed a paravertebral heterogeneous soft tissue mass along the spinal nerve at the L2-L3 level with strong FDG uptake, and a nodule with increased FDG uptake in the lateral lobe of the left liver.

**Diagnosis:**

**A** puncture biopsy of the L3 bony destruction and surrounding soft tissue mass was performed. The final diagnosis was spinal MS with hepatic metastasis.

**Interventions:**

The patient underwent 6 courses of systemic chemotherapy.

**Outcomes:**

The patient did not receive further treatment for half a year after the end of chemotherapy and recovered well.

**Lessons:**

Unlike conventional schwannomas, which are completely benign, MS has an unpredictable prognosis. It is thought to have low malignant potential, and the malignant type tends to metastasize. FDG PET/CT has a unique and important value in the differential diagnosis of benign and malignant lesions, in detecting occult metastases, monitoring the treatment response, and assessing the prognosis of MS.

## 1 Introduction

Melanotic schwannoma (MS) is an unusual variant of a nerve sheath neoplasm that accounts for less than 1% of all primary peripheral nerve sheath tumors. MS was first reported by Millar in 1932, and most often occurs in the spinal nerve root and sympathetic nerve trunk.^[1–5]^ MS occurs at a younger age than conventional schwannoma, without gender predilection, and it is more common in patients aged 30 to 40 years.^[6,7]^ Unlike malignant melanomas, MS has rare mitosis. If there is obvious nuclear atypia, more mitosis, obvious necrosis, or even metastasis, it is considered malignant. Immunohistochemical studies showed that this special nerve sheath tumor strongly expressed S-100, leu-7, HMB-45, and vimentin.^[8–10]^MS has obvious characteristics on magnetic resonance imaging (MRI), but FDG PET/CT has a unique value in detecting malignant MS lesions.^[11,12]^ Only 4 cases of MS with hepatic metastasis have been reported in the literature, and this is the fifth case.^[5,13]^ Malignant MS can show different degrees of local invasion, local recurrence, and distant metastasis.^[5,14,15]^

## 2 Case presentation

A 29-year-old woman with a 1-day history of backache was admitted to our hospital. Physical examination revealed mild weakness and hypermyotonia in the right leg. Lumbar CT revealed bony destruction in the L3 vertebral body and its right adnexa, with a slightly high-density extraspinal soft tissue mass formation. A slight increase in CA724 to 11.29 U/ml was observed, while the other tumor indicators were in the normal range.

**Figure 1:**
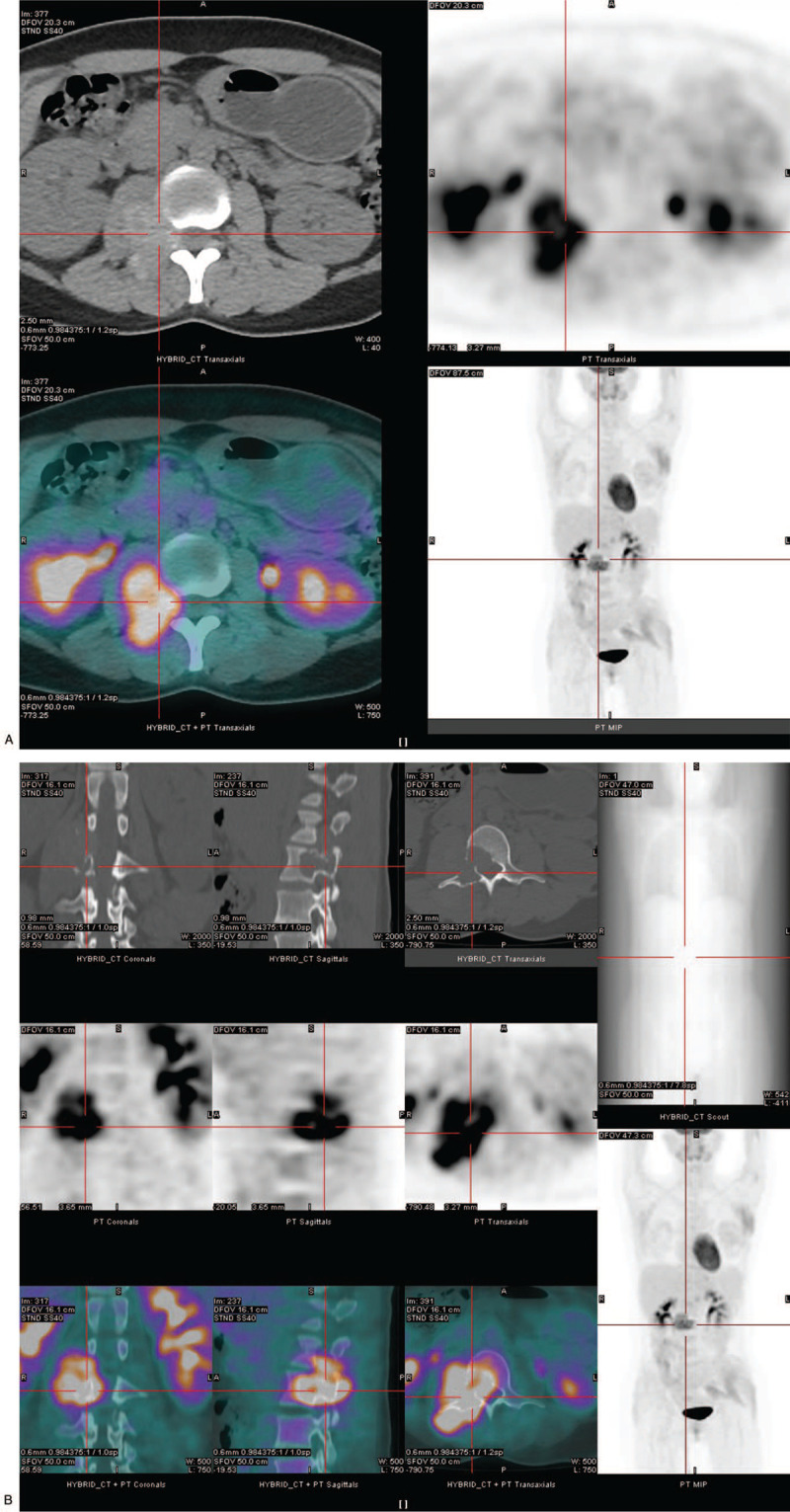
(A). In PET/CT axial series, extraspinal tumor with strong FDG uptake from the spinal canal through the neural foramen was seen. (B). Three-dimensional PET/CT images show the accompanying bone destruction in the L3 vertebral body and its right adnexa.

**Figure 2:**
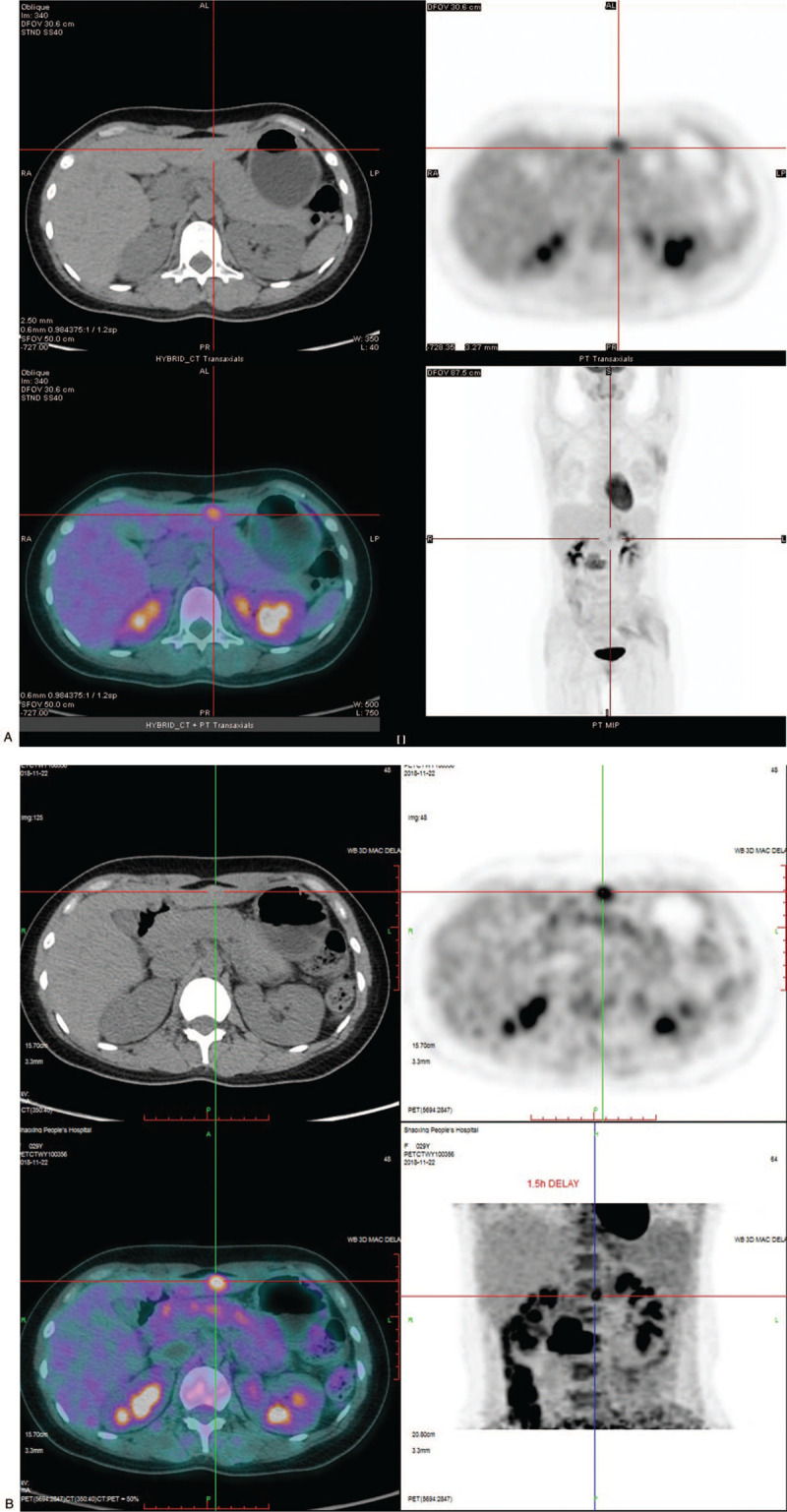
(A). Metastatic hepatic nodule in left lateral lobe with increased FDG uptake on PET/CT, and SUVmax of about 2.98. (B). The delayed PET/CT imaging showed a further increase in SUVmax to 3.79, and the FDG retention index of this hepatic nodule was about 27.2%.

Subsequently, whole-body PET/CT examination was performed. The imaging showed a 43×31 mm paravertebral heterogeneous soft tissue mass along the spinal nerve at the L2-L3 level (Fig. 1a), with strong FDG uptake, accompanied by bone destruction of the L3 vertebral body and its right adnexa (Fig. 1b). The maximum standardized uptake value (SUVmax) was about 8.10 and 9.24 on early and delayed imaging, respectively, and the retention index of FDG was about 14.1%. An 18 × 10 mm nodule with increased FDG uptake was found in the lateral lobe of the left liver (Fig. 2a and 2b), with a smooth edge and a prominent anterior margin, which appeared to be consistent with a metastatic nodule. The CT value was approximately 44 HU. The SUVmax of this nodule on early and delayed imaging was approximately 2.98 and 3.79, respectively. The FDG retention index was approximately 27.2%.

**Figure 3:**
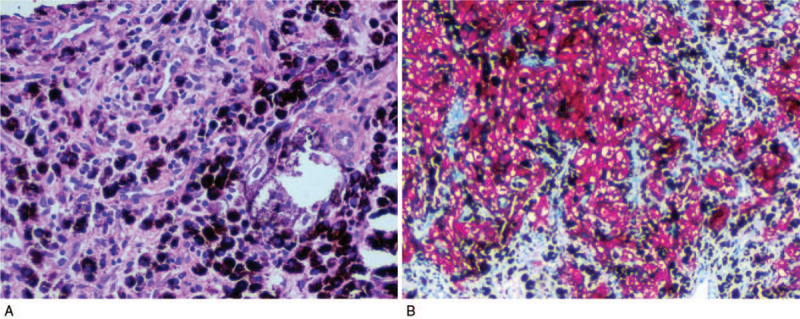
(A). The biopsy specimen showed a markedly pigmented tumor composed of spindle cells with hematoxylin and eosin staining (×100). (B). Cell block preparation from the biopsy specimen immunohistochemically stained with S100 protein (×100), showing positive tumor cells.

A few days later, puncture biopsy of the L3 bony destruction and surrounding soft tissue mass was performed. The smears showed small syncytial tissue fragments and sheets composed of pleomorphic elongated spindle-shaped and ovoid cells containing abundant cytoplasmic pigment associated with numerous pigment-laden macrophages (Fig. 3a). Immunophenotyping revealed positive staining for S-100 (Fig. 3b), vimentin, SOX10, HMB45, MiTF, and Melan-A, but negative staining for EMA and CD57. Thus, the final diagnosis was spinal MS with hepatic metastasis.

The patient underwent 6 cycles of endostar and temozolomide combined chemotherapy, the specific regimen was as followed: Endostar 15 mg intravenous drip, continuous administration from day 1 to 14, and temozolomide 300 mg was administered continuously from day 1 to day 5, with a cycle every 28 days. A whole-body PET/CT examination was performed at another hospital 6 months after the end of chemotherapy, and no active tumor lesions with increased FDG uptake were found in the lumbar spine and liver, suggesting that the patient met the criteria for complete remission. The patient has survived for 1 year since the initial diagnosis.

Ethical approval was not required because this was a case report of the clinical information of the patient. Written informed consent was obtained from the patient for the publication of this case report and accompanying images.

## 3 Discussion

Herein, we report a rare case of spinal melanotic schwannoma (MS) with hepatic metastasis. MS is a rare tumor characterized by melanin secretion originating from Schwann cells, which was first reported by Millar in 1932. ^[1]^ MS accounts for less than 1% of all primary peripheral nerve sheath tumors. It mainly occurs in the paraspinal nerve roots and gastrointestinal tract, but can also be seen in the sympathetic ganglia, spinal cord, acoustic nerve, cerebellum, orbit, choroids, soft tissue, heart, oral cavity, esophageal wall, pancreas, trachea, and bones. The cervical and upper thoracic spinal nerves were the most common sites. ^[2,3–5]^ The most common age of onset was 3 to 84 years, and 30 to 40 years old was the most common. MS has been reported to present at a younger age than conventional schwannoma, without gender predilection. ^[6,7]^

Most MSs are benign or potentially malignant, and the diagnosis mainly depends on pathological examination. Under electron microscopy, epithelial Schwann cells and pigmentation spindle cells are bundle-shaped, interleaved and wheel-shaped, with round and oval nuclei, obvious nucleoli and rare nuclear division. If there is obvious nuclear atypia, more mitosis, obvious necrosis, or even metastasis, it is considered malignant. Immunohistochemical studies showed a staining pattern typical for MS. This special nerve sheath tumor strongly expressed S-100, leu-7, HMB-45, and vimentin, whereas GFAP, EMA, and CK staining were mostly negative.^[8,9]^ In contrast to the typical encapsulation of conventional schwannoma, MS is a circumscribed but unencapsulated tumor, which may reflect the potential of a more aggressive nature such as an invasive growth pattern. Unlike malignant melanomas, which usually have frequent mitosis, MS has rare mitosis. In addition, the histological features of a low proliferative index contribute to the diagnosis of MS rather than malignant melanoma.^[10]^

The lesions of MS were mostly single, with no melanin deposition on the skin. The symptoms are related specifically to the tumor, depending on its location and the rate of growth, and the main manifestations are pain and muscle weakness caused by nerve damage.^[4,5,7]^ MS has obvious characteristics on MRI, with high signal on T1WI and low signal on T2WI due to the paramagnetism effect of melanin, and homogeneous enhancement on post-contrast. ^[6,7,11]^

On PET/CT, the extent of FDG uptake depends on whether the MS lesion is benign or malignant. Yea Eun et al reported a 40-year-old woman with MS, who showed no significant glucose uptake on PET/CT imaging at the initial diagnosis of MS, but 2 years later, her PET/CT images revealed intense FDG uptake in the intercostal mass, but without distant metastasis. Approximately 3 years after the diagnosis of MS, the patient suffered from tumor growth and distant metastasis, and follow-up PET/CT images showed an aggravated state with multiple malignant masses.^[12]^ In our patient, initial PET/CT examination directly revealed a paraspinal mass with significantly increased FDG uptake, and a nodule with abnormal glucose metabolism in the liver, indicating a malignant tumor with distant metastasis, which was confirmed by subsequent needle biopsy. Among various types of liver tumors, malignant tumors such as cholangiocellular carcinoma and poorly differentiated hepatocellular carcinoma also show high FDG uptake on PET/CT. However, considering the age of the patient and the results of the tumor markers, from a monistic point of view, we believe that it is more reasonable to classify this liver nodule as MS metastatic.

One of the important characteristics of tumor tissue is rapid growth and vigorous metabolism, especially the increased rate of glucose glycolysis. Therefore, FDG PET/CT metabolic imaging is one of the most sensitive methods for early diagnosis of malignant tumors. Because of the low incidence of MS and even fewer cases of MS with PET/CT examination, we could not obtain enough information about the PET/CT imaging characteristics of MS patients; therefore, we speculated that PET/CT has the following clinical application values in MS diagnosis:

1. MS is a potentially malignant tumor, and PET/CT can help to differentiate between benign and malignant tumors.
2. FDG PET/CT can clearly show the extent and degree of malignant MS lesions, which provides valuable information for detecting occult metastases.
3. FDG PET/CT can guide the selection of clinical biopsy sites and improve the accuracy of the puncture.
4. FDG PET/CT can be used for efficacy evaluation after chemotherapy or radiotherapy in advanced MS. It can play a very important role in disease staging and monitoring of treatment response.
5. PET/CT can be used for follow-up and predicting the prognosis of MS.

The prognosis of MS is difficult to predict, because it can be locally invasive or metastasize without significant morphological malignancy. FDG PET/CT one-stop examination can detect tumors and determine whether they are malignant based on their glucose metabolism before morphological changes occur.

Vallat-Decouvelaere et al reported the first case of MS with hepatic metastasis in 1987. To date, only 4 cases have been reported, as listed in Table 1.^[5,13]^ In the 1st case, the primary tumor of the spine was completely resected and pathological examination was performed. During the follow-up period, lung, liver and bone metastases occurred, and the patient died 3 years after surgery. The 2nd to 4th cases also had primary lesions in the spine, and the specific diagnosis and treatment are unknown. Two patients had liver metastases only, and 1 patient also had lung and pleural metastases. Two of the 3 patients died during the follow-up period, and 1 patients was still alive during the final follow-up period at 44 months after the initial diagnosis.

**Table 1:** A literature review of five reported cases of melanotic schwannoma with hepatic metastasis, including the present report.

Case No.	Gender	Age (yr)	Primary site	Treatment	Follow-up (mo)/ Outcome	Reference
1	F	45	T6	Surgery	36/DOD	Vallat-Decouvelaere et al^[13]^ (1999)
2	F	23	L4	Unknown	44/AWD	Torres-Mora et al^[5]^ (2014)
3	M	47	L3-L4	Unknown	5/DOD	Torres-Mora et al^[5]^ (2014)
4	F	67	T10	Unknown	10/DOD	Torres-Mora et al^[5]^ (2014)
5	F	29	L2-L3	Chemotherapy	12/AWD	Shen et al (2021, present report)

AWD = alive with disease, DOD = dead of disease, F = female, L = lumbar spine, M = male, T = thoracic spine.

For MS in general, malignant behavior with local invasion occurs in approximately 10% of cases, and local recurrence rates approach one-third in several studies.^[5,14]^ Torres-Mora et al showed that the local recurrence rate of MS was 35%. The rates of distant metastases ranged from 15% in Carney series to 42% in the Torres-Mora series.^[5,15]^ Torres-Mora et al stated that MS should be recognized as a distinctive, malignant neoplasm, rather than simply a schwannoma variant.^[5]^

In summary, MS is a rare neoplasm composed of Schwann cells and melanin pigments. Unlike conventional schwannomas, which are completely benign, MS has an unpredictable prognosis. It is thought to have low malignant potential, and the malignant type tends to metastasize. Herein, we report the PET/CT findings of a case of MS with hepatic metastasis. FDG PET/CT has a unique and important value in the differential diagnosis of benign and malignant lesions, in detecting occult metastases, monitoring the treatment response, and assessing the prognosis of MS. However, because FDG is a non-specific tracer agent, a few benign lesions such as inflammatory granuloma can also show high FDG uptake on PET/CT. In practical applications, we often need to combine a variety of clinical data to make a correct diagnosis.

## Author contributions

**Conceptualization:** Xun-Ze Shen.

**Formal analysis:** Zhou-Ye Lou.

**Resources:** Wei Wang.

**Writing – original draft:** Xun-Ze Shen, Zhou-Ye Lou.

**Writing – review & editing:** Xun-Ze Shen.
